# A Review of Transverse Mode Adaptive Control Based on Photonic Lanterns

**DOI:** 10.3390/mi16121347

**Published:** 2025-11-28

**Authors:** Yao Lu, Zongfu Jiang, Zilun Chen, Zhuruixiang Sun, Tong Liu

**Affiliations:** 1 Space Engineering University, Beijing 101416, China; 2 National Key Laboratory of Space Target Awareness, Beijing 101416, China; 3 College of Advanced Interdisciplinary Studies, National University of Defense Technology, Changsha 410073, China; 4 Department of Aerospace Science and Technology, Space Engineering University, Beijing 101416, China

**Keywords:** photonic lantern, mode control, fiber laser

## Abstract

With the widespread application of fiber laser technology in industries, communications, medical fields, and beyond, the demand for controlling the spatial modes of their output beams has been increasingly growing. Traditional mode control methods are constrained by factors such as device power thresholds, system complexity, and cost, making it difficult to meet the requirements for high-power, high-purity, and rapidly switchable multimode regulation. This paper reviews adaptive mode control technology based on photonic lanterns (PLs). By integrating ideas from adaptive optics and photonics, this technology utilizes photonic lanterns to achieve efficient mode evolution from single-mode to multimode fibers. Combined with optimization algorithms, it enables real-time regulation of input phases, thereby producing stable, high-purity target modes or mode superposition fields at the multimode output end. The paper systematically introduces the structural classifications, propagation characteristics, and fabrication processes of photonic lanterns, as well as the mode evolution mechanisms in different types of photonic lanterns. It elaborates in detail on the structural design, algorithm implementation, and experimental validation of the adaptive control system based on photonic lanterns. Furthermore, it explores the application prospects of this technology in areas such as suppressing transverse mode instability, mode-division multiplexing communications, particle manipulation, and high-resolution spectral measurements. The results demonstrate that the all-fiber adaptive mode control system based on photonic lanterns offers advantages such as compact structure, low loss, fast response, and strong scalability.

## 1. Introduction

T.H. Maiman created the first solid-state laser in history in 1960. Snitzer proposed the use of fiber to produce laser light the next year, and in 1964, he successfully demonstrated the first fiber laser. At the same time that rare-earth ion-doped low-loss fibers and double-cladding fibers were designed and developed, semiconductor laser pumping technology advanced significantly in the 1980s. Over the subsequent decades, fiber laser performance has made tremendous strides, with fiber lasers finding excellent applications in industrial, defense, medical, scientific, and entertainment fields.

As research into fiber lasers has deepened, it has become increasingly recognized that beyond power, wavelength, and polarization, the spatial distribution characteristics of fiber lasers, particularly transverse mode characteristics, are equally important factors determining the practical application value of fiber lasers. Transverse modes are defined as stable field distributions existing in the transverse plane perpendicular to the propagation direction. Different transverse modes possess different application potential: for example, fundamental modes with excellent beam quality and Gaussian intensity distribution can be applied to high-energy laser systems for industrial production and defense applications [1]; OAM modes with helical phase distribution and carrying orbital angular momentum can realize particle manipulation and super-resolution imaging [2,3]. In addition, orthogonality between modes allows them to be multiplexed or coded as new data stream carriers to increase the capacity and efficiency of the communication system [4].

Therefore, how to obtain a stable mode field (or mode superposition field) on demand and how to realize mode control efficiently have become popular research directions in the field of fiber lasers.

Current fiber laser mode control schemes mainly originate from adaptive optics and photonics concepts.

Adaptive optics-based schemes primarily include the following:

(1) adaptive wave-front correction performed on the single aperture fiber laser output, with the modulator selected from Deformable Mirrors [5], liquid crystal spatial light modulators (LC-SLM) [6], polarization controllers, or lensless on-chip microscopy arrays [7] and other devices; (2) adaptive control of the coherent beam combination from fiber laser arrays [8], controlling the phase, intensity, polarization, tilt, and other parameters of each aperture output beam; and (3) enhancement of the loss of non-target modes through adaptive control of the multi-point bending and squeezing [9] of fibers, thereby selecting target modes.

Photonics-based mode control schemes primarily include the following:

(1) designing specialty fibers (such as tapered fibers [10], chirally-coupled-core fibers [11], photonic crystal fibers [12], leakage channel fibers [13], photonic bandgap fibers [14], multi-trench rod-type fibers [15], etc.) to achieve intra-fiber mode filtering; (2) designing long-period fiber gratings to achieve intra-fiber mode conversion [16]; (3) designing fiber couplers [17] to achieve intra-fiber mode selection; and (4) designing metasurface devices/chips at fiber output facets [18] or within fiber laser cavities to achieve mode conversion.

However, all of these mode control schemes are limited by one or more factors, including but not limited to the following: low power threshold of key devices, large system losses, difficult thermal management, high process costs, and system complexity. Facing practical application demands for higher power, higher mode purity, faster mode switching speeds, and more tunable mode numbers, there is an urgent need to find a new implementation pathway for fiber laser mode control. Photonic lantern-based mode adaptive control technology has emerged in response to this need [19]. This technology simultaneously integrates both adaptive optics and photonics concepts, with the core device—the photonic lantern—serving as a novel all-fiber “mode converter” [20] that enables efficient evolution of fundamental mode beams in single-mode fiber (SMF) bundles into multimode beams in a single multimode fiber (MMF). Combined with adaptive optics technology for active control of phases at the single-mode end, stable and controllable mode fields (or mode superposition fields) can ultimately be output at the multimode end.

## 2. Mode Converter: Photonic Lantern

Photonics is defined as the science of shaping the flow of light beams. Applications for photonics have covered various light operations, including light transmission, filtering, noise suppression, interferometry, and more. Common photonic devices include fiber Bragg gratings, fiber couplers, optical circulators, beam shapers, optical switches, etc. However, the performance of these devices often only works effectively in single-mode fiber systems [21].

**Figure 1:**
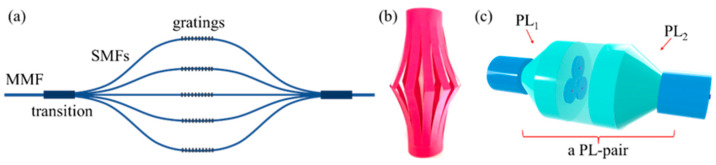
Schematic diagram of photonic lantern structure. (**a**) schematic diagram of the PL; (**b**) paper lantern in daily life; (**c**) a PL-pair.

Driven by application demands in the astronomical field, in 2005, J. Bland-Hawthorn proposed the idea of “using multimode fibers to achieve single-mode fiber Bragg grating filtering performance” during a visit to the University of Bath. Inspired by this, S. G. Leon-Saval, T. A. Birks, and others designed a novel all-fiber mode converter—the photonic lantern (PL) [22]. As shown in the schematic diagram in Figure 1a, it achieves light coupling and evolution through a gradual taper transition region between one multimode fiber (MMF) and multiple single-mode fibers (SMFs), realizing bidirectional lossless transmission between MMF and SMFs. Simultaneously, photonic devices such as fiber Bragg gratings can be inscribed on the single-mode fiber bundle to achieve filtering, providing the possibility for various photonics-based light operations in multimode fiber systems. For SMFs, since only one mode exists, the optical energy is highly concentrated at the core center. When light is reflected by the grating, the photothermal heating is also localized in this central region. This symmetric and concentrated heat source enables highly uniform and efficient radial heat conduction to the cladding and coating layers, ultimately dissipating into the surrounding environment, which facilitates efficient thermal management. Similarly, integrating other photonic devices onto SMFs is feasible, ensuring that the power threshold of photonic lanterns can be significantly high. Due to the structural appearance of this mode converter being similar to decorative lanterns used for illumination in daily life (as shown in Figure 1b), it was named the “photonic lantern.” In subsequent specific research and applications, it has become customary to refer to one MMF-SMFs transition structure as one photonic lantern (PL), and an entire symmetric MMF-SMFs-MMF structure as a photonic lantern pair (PL-pair), as shown in Figure 1c.

As pioneers in the photonic lantern field, T. A. Birks from the University of Bath and S. G. Leon-Saval from the University of Sydney not only first provided the structural design of photonic lanterns but also first conducted detailed analysis of their light transmission characteristics. In 2010, they calculated the propagation constants of modes supported by various cross-sections of photonic lanterns based on the beam propagation method (BPM), revealing the most fundamental principles of the mode evolution process [23]. In 2012, Nicolas K. Fontaine and others from Bell Laboratories studied the influence of different geometric arrangements of single-mode fiber bundles on photonic lantern performance and first introduced transmission matrices to describe the light transmission characteristics of photonic lanterns [24]. To more comprehensively analyze the polarization characteristics of light transmission in photonic lanterns, in 2021, S. G. Leon-Saval led his team to calculate the vector transmission matrix of photonic lanterns using nonlinear neural networks [25]. Thus, the theoretical framework for photonic lanterns was essentially established.

**Figure 2:**
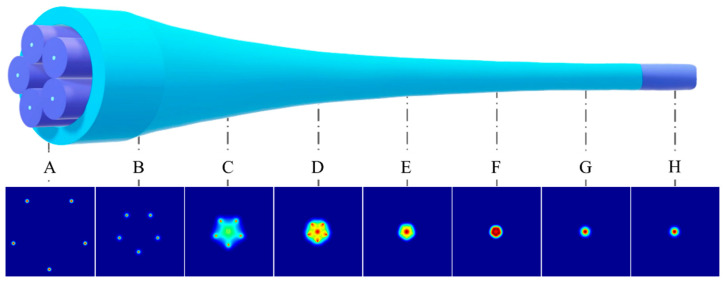
Mode evolution process in a 5 × 1 photonic lantern with equal amplitude and zero phase difference input.

Taking light transmission from the SMFs end to the MMF end as an example, as shown in Figure 2, the specific process of mode evolution is as follows: at one end of the SMFs bundle (point A), light beams exist as fundamental modes with small mode fields propagating independently in separated cores; after entering the taper region (points B, C, and D), as the cores continuously become thinner and closer together, they form a multi-core waveguide structure, causing the light beams to evolve into supermode forms; and at the end of the taper region (points E, F), the modes supported by sufficiently thin and close core groups are essentially the same as the large mode field guided modes in the MMF. This ensures that when light enters the MMF, the mode field overlap integral to both ends of the fusion splice is high, and the light ultimately efficiently evolves into large mode field MMF guided modes or guided mode superposition fields (points G and H).

According to the criterion of whether the single-channel mode evolution results of photonic lanterns exhibit differences, photonic lanterns are divided into two major categories [26]: non-mode-selective photonic lanterns (or mode-group-selective photonic lanterns) and mode-selective photonic lanterns. Non-mode-selective photonic lanterns consist of single-mode fiber bundles composed of completely identical single-mode fibers. When light enters this type of photonic lantern from different signal channels, the mode evolution process exhibits good symmetry, and the output light field obtained at the multimode end is generally a superposition field containing multiple modes. Mode-selective photonic lanterns require the use of single-mode fibers with different structural parameters for bundling to ensure that the fundamental modes supported by each single-mode signal channel have different propagation constants [27], thereby causing differential mode evolution and ultimately obtaining different evolution results. Through rigorous design of single-mode fiber core size gradient distributions and the overall geometric structure design of photonic lanterns, this type of photonic lantern can achieve single-channel light transmission with mode evolution output light fields that are high-purity, large mode field single modes.

Both types of photonic lanterns have their respective advantages: mode-selective photonic lanterns can achieve mode selection without relying on any external control means, requiring only changing the input channel for mode switching; non-mode-selective photonic lanterns, due to their single-mode fiber bundles using the same fiber specifications, have advantages mainly reflected in the following aspects: (1) strong channel number scalability, as non-mode-selective photonic lanterns do not require special core diameter size gradient distributions for single-mode fiber bundles; (2) low fabrication process difficulty, as single-mode fiber bundle assembly is easy, generally achieved by arranging identical single-mode fibers in layered close-packed regular polygonal arrangements; and (3) when working with control systems, non-mode-selective photonic lanterns, due to their good symmetry, can make control algorithm convergence faster and precisely achieve control of mode superposition fields.

According to waveguide theory, the single-mode fiber core bundle taper region of photonic lanterns is an irregular waveguide with longitudinally non-uniform refractive index distribution. The main characteristic of irregular waveguides is that strictly defined modes do not exist, i.e., there are no analytical solutions in the form of *e*(*x*,*y*)ּ *e*^(−*iβz*). In this case, we can find a regular waveguide segment such that the field within the irregular waveguide can be expanded as a series sum of modes of this regular waveguide. This expansion includes not only the discrete sum of guided modes but also the continuous sum of radiation modes (where radiation modes are radial standing wave distributions formed by the superposition of incident and reflected light outside the fiber core).

The focus of studying photonic lantern mode evolution processes is the calculation of eigenmodes including guided modes and radiation modes, and the calculation of mode superposition and coupling between them. Methods such as the beam propagation method (BPM) [28] or mode expansion propagation method [29] can be used to numerically simulate the light propagation process in photonic lanterns, obtaining the one-to-one correspondence relationship between input and output light fields of photonic lanterns. Mathematically, the relationship between input and output light fields of photonic lanterns can be compactly expressed as follows:

(1) u=M⋅v,

where *M* is the transmission matrix of the photonic lantern, *v* is a column vector describing the light field information at the single-mode end of the photonic lantern, *v^T^ =* [*v*_1_*e^iθ^*_1_, *v*_2_*e^iθ^*_2_, *v*_3_*e^iθ^*_3_,…, *v_j_e^iθj^*,…] (where *v_j_* and *θ_j_* represent the amplitude and phase of the fundamental mode light field input from the *j*-th single-mode fiber, respectively), and *u* is a column vector describing the light field information at the multimode end of the photonic lantern, *u^T^ =* [*u*_1_*e^iφ^*_1_, *u*_2_*e^iφ^*_2_, *u*_3_*e^iφ^*_3_,…, *u_k_e^iφk^*,…] (where *u*_k_ and *φ*_k_ represent the amplitude and phase of the *k*-th eigenmode in the output superposition field, respectively).

For example, when using linearly polarized mode (LP mode) sequences as eigenmode sequences, the transmission matrix of an ideal 3 × 1 mode-selective photonic lantern is Equation (2), while that of a 3 × 1 non-mode-selective photonic lantern is Equation (3):

(2) $$M_{LP,3\times 1MSPL}^{ideal}=\left[\begin{array}{ccc} 1 & 0 & 0\\ 0 & 0 & 1\cdot e^{i(0.2\pi )}\\ 0 & 1\cdot e^{i(-0.4\pi )} & 0 \end{array}\right],$$

(3) $$M_{LP,3\times 1MGPL}^{ideal}=\left[\begin{array}{ccc} \frac{1}{\sqrt{3}} & \frac{1}{\sqrt{3}} & \frac{1}{\sqrt{3}}\\ \frac{1}{\sqrt{6}} & \frac{1}{\sqrt{6}} & \frac{2}{\sqrt{6}}e^{i\pi }\\ \frac{1}{\sqrt{2}} & \frac{1}{\sqrt{2}}e^{-i\pi } & 0 \end{array}\right],$$

When the taper region of the photonic lantern is sufficiently long and satisfies the adiabatic approximation condition, the photonic lantern can be viewed as a linear optical device, and its transmission matrix is invertible. Therefore, to obtain a specific mode at the multimode fiber output end of the photonic lantern, one only needs to set the corresponding term for that mode in column vector *u* to 1 and other terms to 0, and then calculate the required input light field combination according to Equation (1):

(4) $$v_{\mathrm{target}\;\mathrm{mode}}=M^{-1}\cdot u_{\mathrm{target}\;\mathrm{mode}},$$

As the core device for achieving mode evolution between single-mode fiber systems and multimode fiber systems, the performance and reliability of photonic lanterns greatly affect mode control effectiveness. Therefore, many researchers are dedicated to continuously optimizing photonic lantern fabrication processes. T. A. Birks categorized photonic lantern fabrication schemes into three types: direct ultrafast laser inscription, tapering multicore fiber, and tapering SMF-tube bundle method. Among these, the tapering SMF-tube bundle method is most favored.

**Figure 3:**
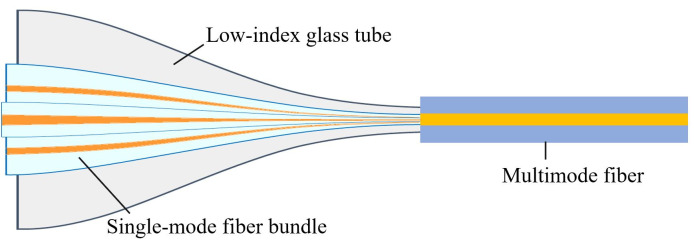
Side view of the structure of the photonic lantern fabricated by the tapering SMF-tube bundle method.

Taking a three-channel example, the structural side view of a tapering SMF-tube bundle photonic lantern is shown in Figure 3. Its fabrication steps are as follows: first, a group of separated single-mode fiber bundles are closely arranged and inserted into a low-index glass tube, then simultaneously fused and tapered. As the transverse dimensions decrease, the fiber cores in the single-mode fiber bundle continuously approach each other and become thinner, forming the core of a new waveguide structure, while the low-index glass tube becomes the new cladding. Second, the tube-single-mode fiber bundle is cut at the waist region (the area with the smallest diameter). Finally, the waist region end face of the tube-single-mode fiber bundle is fusion-spliced with a matching multimode fiber. This method produces photonic lanterns with separated single-mode end fibers, making it easy to achieve light field control at the single-mode end [30]. Additionally, the raw materials for making such photonic lanterns are common step-index fibers, ensuring good compatibility with fiber laser seed sources, amplifier stages, and other fiber systems.

Due to practical factors such as small fiber cross-sectional dimensions and reduced waveguide structure strength after fusion tapering, process details have a significant impact on photonic lantern yield and performance. Key process details requiring optimization include the following: device cleanliness, single-mode fiber bundle assembly techniques, fusion tapering parameters, and fusion splice alignment [31]. In 2009, Noordegraaf et al. from Denmark fabricated the first high-efficiency, low-loss seven-channel non-mode-selective photonic lantern based on the tube fusion tapering method [32]. In 2015, Velazquez-Benitez et al. from the University of Central Florida completed the design and fabrication of a six-channel mode-selective photonic lantern using the tube method [27], achieving insertion loss of only 0.2–0.3 dB and mode purity exceeding 9 dB for all channels. In 2021, John J. Davenport et al. from Germany conducted detailed studies on the influence of bundling conditions on photonic lantern performance in tube method fabrication schemes and completed the fabrication and testing of multi-channel (7, 19, and 37 channels) non-mode-selective photonic lanterns [33].

## 3. Transverse Mode Adaptive Control System

The basic principle of adaptive optics is to compensate the transmitted signal based on the detected received signal, belonging to feedback-type active control systems, roughly divided into two categories: conjugate systems and optimized systems. Conjugate systems require real-time detection of actual input signals and calculation of the difference between them and ideal input signals reverse-calculated from target output signals; then, they compensate for this difference. If photonic lanterns are applied in such systems, it would be necessary to interrupt the input end and introduce a section of free-space optical path to detect input signals, which would undermine the advantages of photonic lanterns as all-fiber devices.

Optimized adaptive control systems calculate a certain evaluation function at the output signal end in real-time to reflect system compensation performance, using optimization algorithms to help the input end search for appropriate modulation signals so that the evaluation function at the output end converges to a unique extremum. Optimized adaptive control systems avoid measurement of input end signals and calculation processes for modulation signals, simplifying experimental optical path complexity while providing better scalability for system channel numbers.

**Figure 4:**
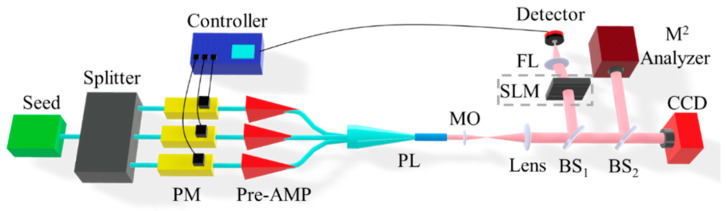
Schematic diagram of mode adaptive control system based on 3 × 1 photonic lantern.

Borrowing from the optimized structure, a photonic lantern-based mode adaptive control system is designed, as shown in Figure 4: a single-frequency linearly polarized seed laser travels along single-mode fibers, passes through a splitter, then through phase modulators (PM) and pre-amplifiers (Pre-AMP) on each single-mode polarization-maintaining line, and is injected into the single-mode fiber end of the photonic lantern, outputting at the multimode fiber end of the photonic lantern. A PM is used for on-line phase modulation of each path under algorithm control, while Pre-AMP is used for off-line adjustment of input power for each path of the photonic lantern.

In the external optical path, a microobjective (MO) and a large focal length lens combination form a 4F system (4F Fourier lens system) to magnify the near-field pattern size at the photonic lantern output end. Beam Splitter 1 (BS1) splits out one output signal path to the detector, providing an evaluation function for the controller. When necessary, the output signal can pass through a spatial light modulator (SLM) for optical filtering preprocessing. Beam Splitter 2 (BS2) splits the output beam to a CCD camera and M^2^ analyzer for near-field spot detection and beam quality analysis of control effects.

The mode evolution process in photonic lanterns stems from continuous mode superposition and coupling of light within them. The occurrence of these two interactions depends on the coherence between input light fields of each path in the photonic lantern. The fundamental mode beams at each path of the photonic lantern input end shown in Figure 4 are emitted from the same single-frequency seed source, with essentially consistent beam frequencies between paths and long coherence length. Additionally, all devices before the photonic lantern input end are linearly polarized devices connected by polarization-maintaining fibers. Therefore, ensuring essentially the same optical path length between paths can satisfy coherence conditions.

Phase noise commonly exists in practical fiber laser systems, where phase noise introduced by environmental disturbances generally has characteristic frequencies below the kilohertz level, and phase noise introduced by gain media themselves generally has characteristic frequencies below the hundred-hertz level. To ensure that the mode adaptive control system can achieve modulation and locking of input phases for each path, the system’s control bandwidth must be higher than the highest characteristic frequency of phase noise. Lithium niobate (LiNbO_3_) crystal phase modulators are generally chosen as modulation devices, offering advantages such as high response frequency (GHz level) and half-wave voltage that does not drift with modulation frequency. Additionally, many optimization algorithms can easily generate disturbance signals of several hundred kHz. Therefore, as long as suitable optimization algorithms are found and parameters are tuned to enable fast convergence (within 100 loops) and achieve control bandwidth above the kHz level, phase modulation and locking can be realized.

Commonly used optimization algorithms include the following: dithering algorithm, genetic algorithm, hill climbing algorithm, simulated annealing algorithm, and Stochastic Parallel Gradient Descent (SPGD) algorithm. Their working principles determine the response time for single-step operations and the number of iterations required for convergence, thereby determining control bandwidth. The SPGD algorithm is an efficient algorithm that performs gradient estimation each time modulation signals are updated [34]. The modulation signal iteration formula for this algorithm is as follows: ***u***^(^*^m^*^+1)^ = ***u***^(^*^m^*^)^ + *γδ**u***^(^*^m^*^)^∆*J*^(^*^m^*^)^, where *m* is the current iteration number, *γ* is the gain coefficient, *δ**u***^(^*^m^*^)^ is the random disturbance voltage magnitude in the m-th iteration, and Δ*J* is the evaluation function change amount, providing gradient information for iteration. The introduction of this parameter reduces the number of iterations required for evaluation function convergence, thereby improving overall system control bandwidth. Additionally, through appropriate evaluation function selection and further parameter optimization, the SPGD algorithm also offers advantages such as strong system scalability and high power fluctuation tolerance.

Without loss of generality, taking a 3 × 1 non-mode-selective photonic lantern achieving mode control at a 30 μm core diameter multimode fiber output end as an example, numerical simulation of the execution process of the photonic lantern-based mode control system is performed. The evaluation function *J* is taken as the power in bucket (PIB) of the near-field spot at the multimode output end of the photonic lantern, with the specific position of the “bucket” determined according to different mode control objectives. Through parameter optimization, it is determined that when disturbance voltage *δ* = 0.07 and gain coefficient *γ* = 200, the average convergence speed of the evaluation function is fastest.

**Figure 5:**
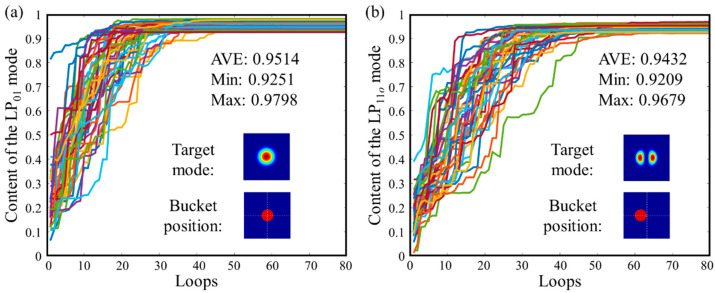
Using the SPGD algorithm, and achieving (**a**) LP_01_ mode and (**b**) LP_11_*_o_* mode control based on a 3 × 1 non-mode-selective photonic lantern.

Figure 5 shows numerical simulation results for achieving LP_01_ mode or LP_11_*_o_* mode output after executing control algorithms with 50 random fundamental mode combinations as initial inputs. During calculations, initial phases for each path are set as random phases within the 0 to 2π range, and input power for each path at the single-mode fiber end randomly fluctuates by 10% based on ideal values calculated according to Equation (4). Figure 5a shows the change in LP_01_ mode content obtained through real-time mode decomposition of output spots when LP_01_ mode is the control target. In this case, the “bucket” is selected at the spot center (position shown by red dot in the figure). The algorithm basically completes convergence within 50 loop cycles. After convergence, the output light field remains stable, with an average convergence value of LP_01_ mode content of 0.9514 across 50 calculations, minimum value of 0.9251, and maximum value of 0.9798. Figure 5b shows control effects when LP_11_*_o_* mode is the control target. In this case, the “bucket” is selected at a position away from the spot center. The algorithm convergence speed has a slight decrease, basically completing convergence within 60 loop cycles. After convergence, the average convergence value of LP_11_*_o_* mode content is 0.9432.

The above results show that after selecting appropriate control parameters, even under conditions of 10% power fluctuation at each input path, the SPGD algorithm can still efficiently complete phase locking and achieve stable, high-purity target mode output.

**Figure 6:**
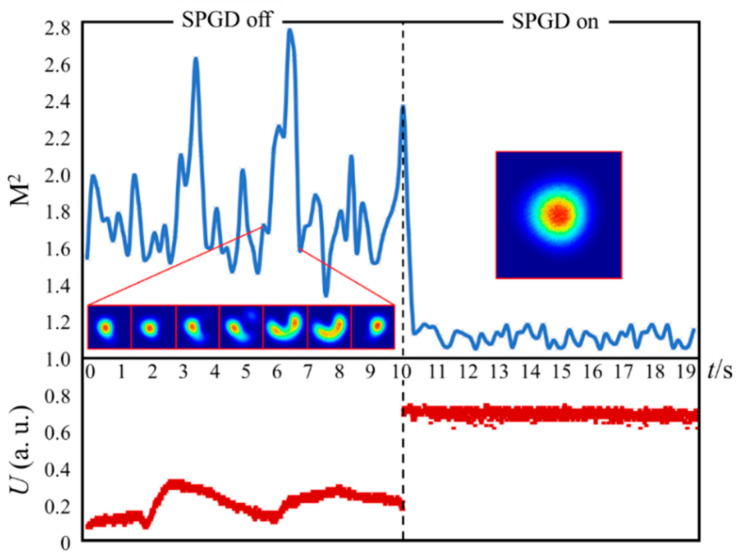
Changes in output spot, M^2^ factor, and power in bucket before and after algorithm execution when fundamental mode is the control target.

Building the mode adaptive control system shown in Figure 4, Figure 6 shows the changes in near-field light intensity distribution, beam quality, and oscilloscope-displayed detector voltage signals of measured output beams before and after control algorithm closed-loop operation when the fundamental mode is the control target. When the system is open-loop (SPGD off), the near-field spot morphology continuously changes and is in a multimode superposition state, the M^2^ factor continuously fluctuates with poor beam quality, and the detector voltage signal continuously oscillates with low amplitude. When the system is closed-loop (SPGD on), the spot locks to a quasi-fundamental mode form (fundamental mode content exceeding 0.95) in a very short time, the M^2^ factor slightly fluctuates below 1.2, and the oscilloscope shows that the voltage signal amplitude is high and remains stable.

**Figure 7:**
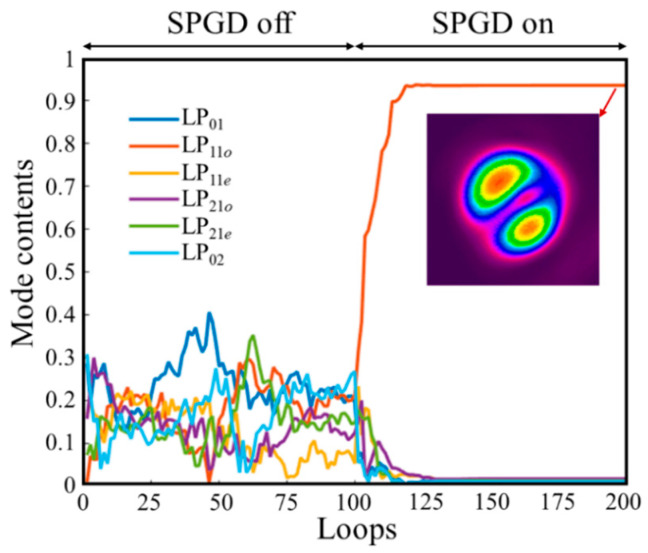
Changes in output beam mode proportion when LP_11_*_o_* mode is the control target and near-field light intensity distribution after closed-loop system operation.

Figure 7 shows the changes in mode proportion of measured output beams before and after control algorithm closed-loop operation when LP_11_*_o_* mode is the control target. In this case, the relative positions between the bucket and spot centroid and the bucket size are changed by adjusting the positions of the detector and aperture and their distances from the lens. Experimental results show that when the system is open-loop, the mode content of output beams continuously changes with energy conversion between modes, while when the system is closed-loop, the output spot stabilizes in a quasi- LP_11_*_o_* mode form with LP_11_*_o_* mode proportion of approximately 0.924.

## 4. Application of All-Fiber Mode Control Systems

**Figure 8:**
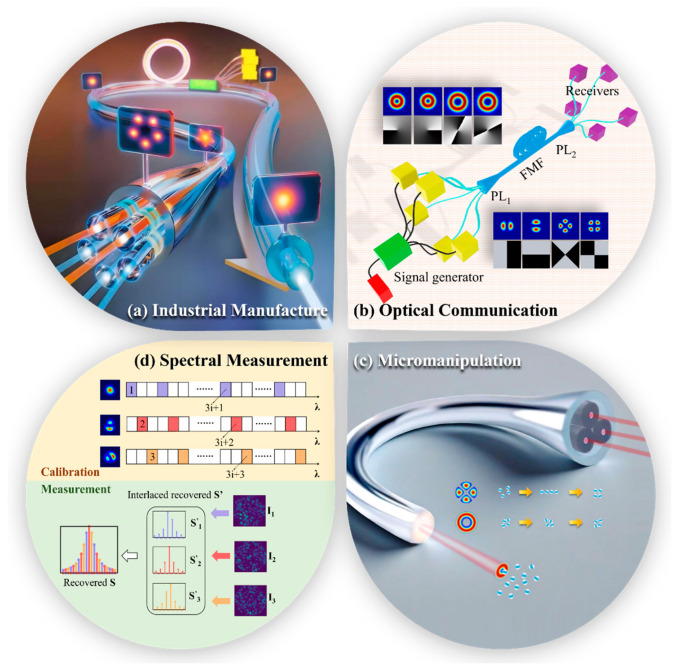
Schematic diagram of application scenarios for photonic lantern-based mode control.

Photonic lantern-based mode control systems achieve all-fiber implementation and are expected to meet practical application demands for higher power, higher mode purity, faster mode switching speeds, and more tunable mode numbers. They have broad application prospects in industrial manufacturing, optical fiber communications, and frontier exploration fields, as shown in Figure 8.

### 4.1. Industrial Manufacturing

In 2000, Beck et al., to improve laser processing precision, controllable dimensions, and remote operation capabilities, coupled laser light emitted by Nd: YAG lasers with fibers and first used fundamental mode beam output from fiber end faces for material processing [35]. Since then, fiber lasers have gradually developed into indispensable tools in the industrial manufacturing field. According to the basic principles of light-matter interaction, the key for fiber lasers to meet application demands in industrial manufacturing and other high-energy laser fields (such as directed energy technology and gravitational wave detection) is using fiber lasers to emit high-power, mode-stable light beams.

In 2008, Dawson et al. theoretically calculated [36] and predicted that the ultimate output power limit for single-frequency fiber lasers is 1.86 kW, and for broadband fiber lasers is 36.6 kW. However, this prediction was based on the assumption that fiber mode field size is unlimited. To achieve ultimate power output, implementation in 90 μm core diameter or even larger fibers would be required, making it difficult to guarantee output beam mode stability. Experimentally, in 2011, Eidam et al. discovered that fiber laser beam quality indeed degrades due to the influence of transverse mode instability (TMI) effects [37], specifically manifesting as continuous energy coupling between multiple modes in output beams.

Addressing this problem, in 2017, Lincoln Laboratory achieved 1.2 kW stable output in a 25 μm core diameter ytterbium-doped fiber amplifier through active control of input beam phases in a 3 × 1 non-mode-selective photonic lantern [38], experimentally verifying that this scheme can improve system TMI threshold.

TMI is the result of thermally induced nonlinear effects [39]. Fundamental modes and higher-order modes propagate along active fibers with different phase velocities, forming periodic light intensity distributions (mode interference patterns, MIPs) during this process. This causes periodic thermal load distributions on active fibers, producing thermally induced refractive index changes (RIGs), ultimately inducing energy exchange between different modes in fibers.

The mechanism by which photonic lantern-based mode control systems suppress TMI is actively generating controllable mode superposition fields and using them as seeds for fiber laser main amplification stages. These mode superposition fields, after entering active fibers, can produce appropriate MIP to block TMI occurrence or terminate TMI occurrence. Blocking TMI occurrence means that during the process of system output power continuously increasing from below the inherent TMI threshold to exceeding the TMI threshold, using photonic lantern mode control systems to actively generate fast controllable mode superposition fields cancels out MIPs produced by defects and environmental disturbances in active fibers, preventing them from having time to produce thermal effects and preventing RIG formation. Terminating TMI refers to when fiber laser systems have already experienced TMI, generating MIPs conjugated to inherent MIPs so that their thermal effects “wash out” existing RIGs, ultimately achieving TMI termination.

**Figure 9:**
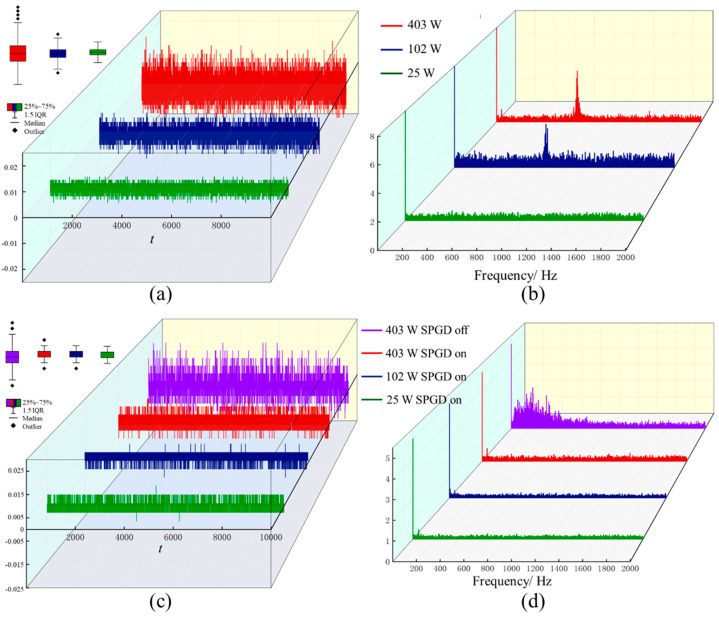
Changes in time-domain and spectral characteristics of output beams from ytterbium-doped fiber amplifiers with conventional/photonic lantern seed sources at different output powers [40]. (**a**,**c**) time-domain signal; (**b**,**d**) spectral signal.

Taking a 5 × 1 photonic lantern-based all-fiber mode control system as an example, its output beam is used as seed for a 42 μm core diameter ytterbium-doped fiber amplifier to verify its effect in blocking TMI occurrence. The gain fiber selected for the main amplification stage is NA = 0.075 non-polarization-maintaining fiber with absorption coefficient of approximately 18 dB/m @ 976 nm. When this amplifier uses conventional seed sources, the TMI power threshold *P_thr_* is approximately 100 W, meaning that when output power exceeds 100 W, the time-domain signal dispersion of output beam power in bucket significantly increases (as shown in Figure 9a), with characteristic peaks beginning to appear around 800 Hz in the corresponding Fourier spectrum, and as power continues to increase, characteristic peaks show trends of narrowing width and increasing peak values (as shown in Figure 9b).

When using output beams from the photonic lantern all-fiber mode control system as seed sources: under closed-loop control algorithm conditions, continuously increasing pump power, when output power is below, above, or even four times *P_thr_*, time-domain signal dispersion remains small (as shown by green, blue, and red curves in Figure 9c), with only occasional outliers appearing as power increases. Simultaneously, no characteristic peaks appear in corresponding Fourier spectrum plots (as shown by green, blue, and red curves in Figure 9d). If the control algorithm is turned off when output power is above *P_thr_*, as shown by purple curves in Figure 9c,d, the time-domain signal interquartile range increases significantly with many outliers, characteristic peaks with low peak values and large widths appear in Fourier spectra (frequency coverage 0–600 Hz), average output power decreases significantly, gain fiber temperature rises rapidly, and sustained light output becomes impossible.

Using M^2^ analyzers to compare beam quality at *P_out_* = 367 W, measurements show that when using conventional seed sources, due to TMI occurrence, output spots are lobed and continuously changing, with *M_x_*^2^ = 2.646 and *M_y_*^2^ = 2.503, indicating poor beam quality. When using photonic lantern mode control systems as seed sources under closed-loop system conditions, output spots exhibit stable quasi-fundamental mode forms with *M_x_*^2^ = 1.762 and *M_y_*^2^ = 1.567, indicating good beam quality. The above experimental results prove that photonic lantern mode control systems not only excellently suppress TMI but also significantly improve beam quality, well meeting demands for high-power, high-brightness, large mode field laser output in industrial manufacturing and other high-energy laser systems.

### 4.2. Optical Fiber Communications

To increase capacity, improve efficiency, and ensure security, optical communication system builders are dedicated to researching various multiplexing techniques or coding techniques to achieve multi-dimensional development and utilization of optical wave resources. Following time domain, wavelength, and polarization of light, the spatial distribution (mode) of light has become the newest (and possibly last) degree of freedom that can be utilized, considered an important handle for continued development of optical communication systems [41].

Photonic lanterns, as adiabatic mode converters, can evolve a group of discrete fundamental mode beams into a fundamental mode or higher-order mode with larger mode field area, and vice versa. This special light transmission characteristic ensures that they can excellently perform multiplexing/demultiplexing or coding/decoding functions in communication systems. Over the past decade, the practical value of photonic lanterns in multiple-input multiple-output (MIMO) networks and optical interconnection networks has been successively verified: In 2012, Nicolas K. Fontaine et al. proposed the concept of using three-channel mode-selective photonic lanterns for mode division multiplexing [42]. In 2014, Uden et al. [43] and Paul Mitchell et al. [44], respectively, designed 21 (3 × 7)-channel and 51 (3 × 19)-channel integrated photonic lanterns and used them in communication systems with three types of mode division multiplexing combined with seven (or nineteen) types of frequency multiplexing, not only improving communication capacity but also proving device reliability in multi-dimensional multiplexing communication technology. In 2021, A. Alarcón et al. from Sweden used photonic lanterns in quantum communication systems, achieving generation, encoding, and detection of three spatial photon states [45].

However, the photonic lanterns selected in the above optical communication systems are basically mode-selective photonic lanterns. The requirements for input fiber core diameter differences in structural design of this type of photonic lantern will undoubtedly become the greatest limiting factor for expanding the number of system mode multiplexable modes and an important source of inter-mode crosstalk. A feasible optimization method is to use non-mode-selective photonic lantern-based mode adaptive control systems for mode division multiplexing or encoding, then use the same photonic lanterns for mode division demultiplexing or decoding, as shown in Figure 8b.

**Figure 10:**
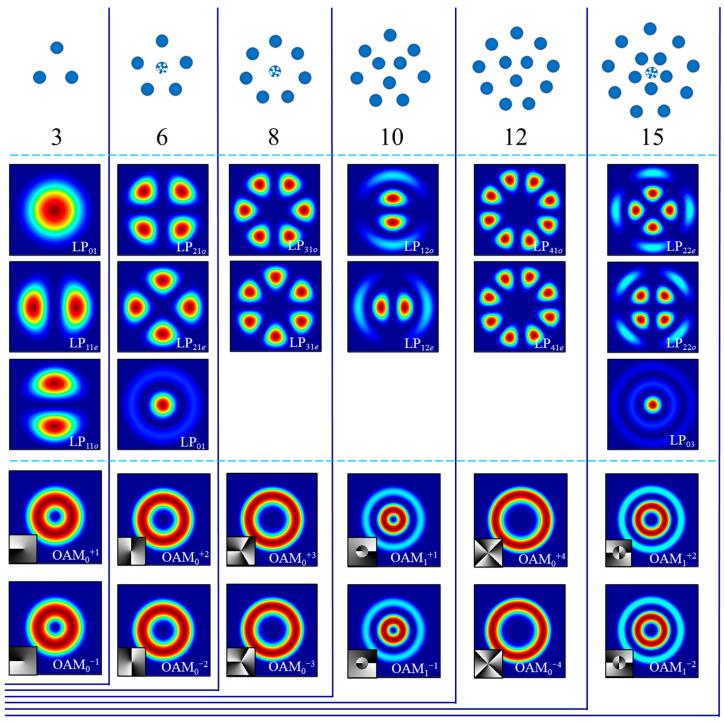
Single-mode fiber bundle arrangements for different channel number photonic lanterns and their mode control ranges.

When using photonic lantern all-fiber mode control systems to generate specific modes or mode superposition combinations, geometric arrangement schemes of single-mode fiber bundles can be optimized to improve control algorithm convergence speed. For LP modes supported by step-index fibers, their morphological patterns are as follows: for LP*_mn_* modes, their spots have 2*m* lobes in the angular direction and *n* − 1 layers in the radial direction, and when *m* > 0, LP*_mn_* modes have two degenerate states. Therefore, corresponding photonic lantern single-mode end fiber core arrangement schemes should be 2*m* + 1 fibers uniformly distributed in regular polygons in the angular direction, with *n* − 1 layers of fibers distributed in the radial direction. Following the above method, Figure 10 shows geometric arrangements of single-mode fiber bundles for different channel number photonic lanterns and their corresponding LP modes.

When two degenerate states of LP*_mn_* modes (m > 0) are linearly superposed with equal amplitudes and phase differences of ±π/2, they can form orbital angular momentum (OAM) modes [46,47]. Their light field distribution *E*(*OAM_p_^l^*) can be written as follows:

(5) $$E({{OAM}_{p}}^{\pm l})=E(LP_{mno})\pm \;e^{i\pi /2}E(LP_{mne}),\;l=m,p=n-2$$

where *l* is the topological charge number, and each photon in the beam carries average orbital angular momentum of *lħ* (*ħ* is Planck’s constant). Therefore, digital encoding can be performed according to different orbital angular momentum numbers carried by OAM modes. The phase expression of OAM modes contains the spiral term *e^ilφ^*, making different OAM modes highly orthogonal to each other, enabling good mode division multiplexing. Particularly, when control targets are OAM mode sequences, the central fiber in 6 × 1, 8 × 1, 15 × 1, … photonic lantern single-mode fiber bundles can be replaced with no-core fibers to improve control efficiency [48].

When photonic lantern-based all-fiber mode control is used for optical fiber communications, the number of tunable modes depends on photonic lantern channel numbers. Currently reported photonic lantern channel numbers have reached several hundred [49], meaning theoretically hundreds of modes can be tuned. Mode switching speed depends on system control bandwidth. Optimization algorithm-based adaptive control systems can easily achieve control bandwidth above 10 kHz, so systems are expected to achieve mode switching speeds on the order of 10 kHz, promising to play irreplaceable roles in optical communication systems.

### 4.3. Frontier Exploration

Photonic lantern-based mode control systems possess advantages of all-fiber implementation, compact structure, low loss, and single-aperture fiber output, enabling applications in many frontier exploration fields.

In 2018, Velázquez-Benítez et al. employed three-channel mode-selective photonic lanterns to achieve single-particle trapping and manipulation. Additionally, they utilized the large-mode-field, switchable linearly-polarized modes output from photonic lanterns to realize the trapping and rotation of double-particles or particle clusters. This approach demonstrated extremely high flexibility at the single-aperture multimode fiber output end [50]. Furthermore, if photonic lantern-based all-fiber mode control systems are used to generate freely switchable higher-order linearly polarized modes carrying gradient forces or OAM modes carrying orbital angular momentum, simultaneous particle trapping, rotation, and transport in multiple planes could be achieved, as shown in Figure 8c.

In 2021, Moraitis et al. developed a novel photonic lantern approach for PolyOculus fiber optic linkages that potentially offers substantial advantages over previously considered free-space optical linkages, including much higher coupling efficiencies [51]. PolyOculus is for producing large-area-equivalent telescopes by using photonic technologies to link modules of multiple semi-autonomous, small, inexpensive, commercial-off-the-shelf telescopes. We can boldly imagine that in the future, introducing adaptive control systems and properly modulating the input ends of photonic lanterns will further improve throughput efficiency and beam quality, resulting in better astronomical observation results.

In 2024, Liang et al. proposed a high-precision all-fiber mode division multiplexing reconstructive spectrometer (RS) [52]. By using photonic lanterns to adjust mode field distribution conditions incident on scattering media, spectra are more comprehensively encoded into multiple speckle patterns. They achieved 2 pm spectral resolution and reconstruction of 2000 spectral channels with significantly reduced recovery errors, as shown in Figure 8d. In the future, by increasing photonic lantern channel numbers and thereby increasing generated mode types, efficient application to higher precision, higher resolution spectral measurement scenarios could be achieved.

## 5. Conclusions

In this paper, we have extensively reviewed the core mode evolution device, adaptive control mechanism, and applications of the transverse mode adaptive control system based on photonic lanterns. Regarding system cost, this all-fiber system employs internal optical path modulation, resulting in a compact system structure. In terms of process complexity, the fabrication difficulty of photonic lanterns is significantly lower compared to that of specialty optical fibers and advanced metasurface devices. In terms of power scalability, the photonic-lantern-based mode control system is an all-fiber system with single-aperture output. It can be efficiently and low-loss fused with common fiber laser systems (especially fiber laser amplification stages), exhibiting excellent power amplification capabilities. We look forward to the rise of new applications, as this system possesses advantages of all-fiber implementation, compact structure, low loss, and single-aperture fiber output.

## Data Availability

The original contributions presented in the study are included in the article; further inquiries can be directed to the corresponding authors.
